# Supplementary material for: Emerin loss of function inhibits MyoD-driven differentiation of human iPSCs into skeletal myotubes
Source: bioRxiv. 2026 Sep 16:2026.09.14.750687. Preprint. [Version 1] doi: 10.64898/2026.09.14.750687 (PMC13596264; doi:10.64898/2026.09.14.750687)
Supplement: Supplementary file 1 [file NIHPP2026.09.14.750687v1-supplement-1.pdf]

bioRxiv preprint doi: https://doi.org/10.64898/2026.09.14.750687; this version posted September 16, 2026. The copyright holder for this preprint (which was not certified by peer review) is the author/funder, who has granted bioRxiv a license to display the preprint in perpetuity. It is made available under a CC-BY 4.0 International license.

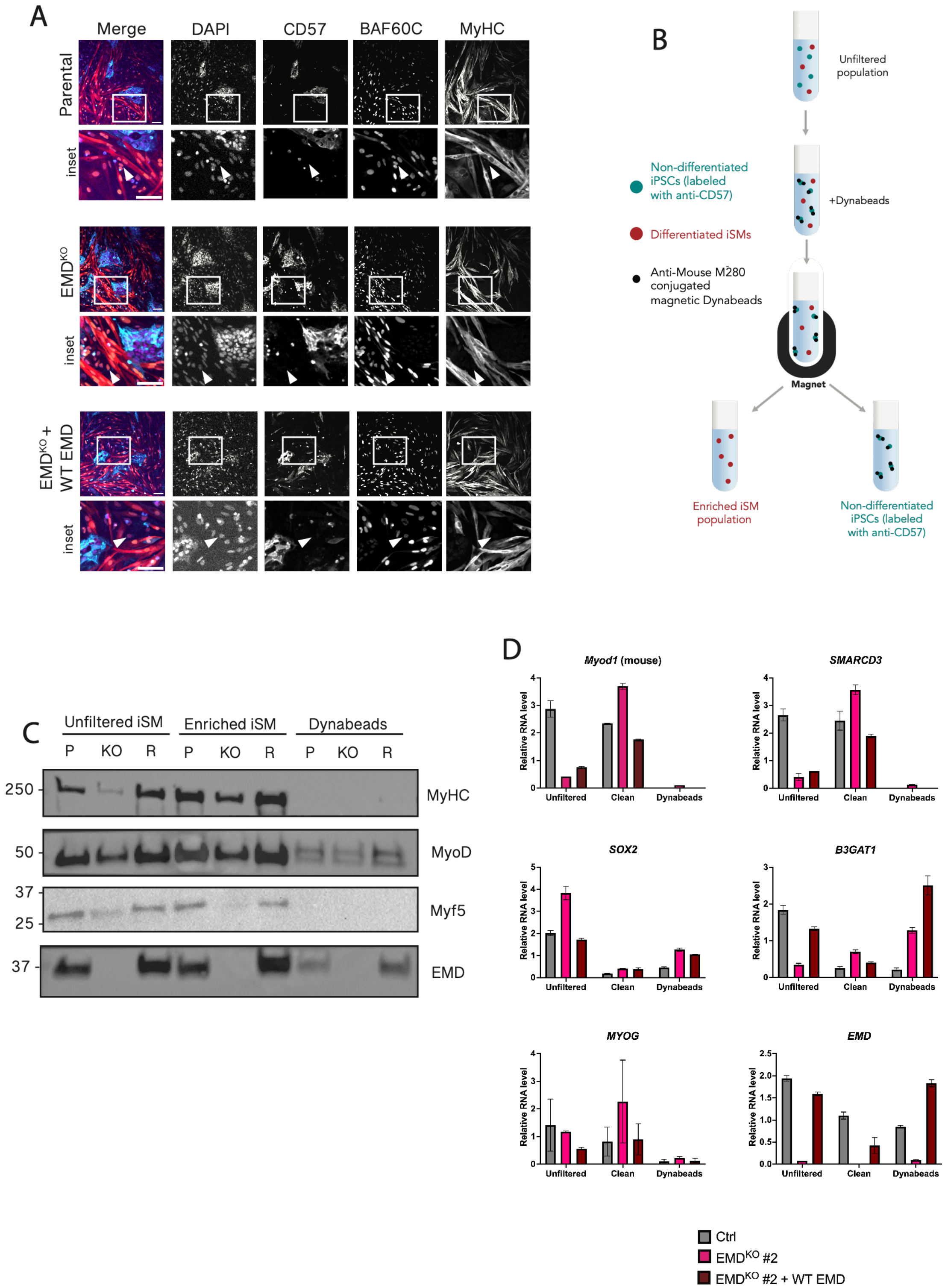

Supplementary Figure 1. Validation of iSM immunodepletion protocol.

(Figure caption continued on the next page.)

bioRxiv preprint doi: https://doi.org/10.64898/2026.09.14.750687; this version posted September 16, 2026. The copyright holder for this preprint (which was not certified by peer review) is the author/funder, who has granted bioRxiv a license to display the preprint in perpetuity. It is made available under a CC-BY 4.0 International license.

(Figure caption continued from the previous page.)

A) Immunofluorescence staining of Day 4 iSMs, co-stained for BAF60C, MyHC, and CD57. White arrows indicate examples of BAF60C+/CD57- nuclei. B) Diagram detailing iSM cleanup protocol. C) Western blot of myogenic factors and emerin in unfiltered and enriched iSM populations, as well as CD57+ cells captured by Dynabeads. P = parental, KO = EMD^{KO} #1, R = EMD^{KO} #1 + WT EMD (Rescue). D) RT-qPCR of select genes in CRISPR control clone, EMD^{KO} #2, and EMD^{KO} #2 + WT EMD Day 6 iSMs, comparing unfiltered, enriched, and Dynabead-bound populations (n = 3 technical replicates per condition). RNA level calculated relative to GAPDH control. Gene transcripts quantified: *Myod1* (mouse MyoD transgene), *SMARCD3* (encoding BAF60C), *SOX2*, *B3GAT1* (encoding CD57 synthase), *MYOG* (encoding myogenin), and *EMD*. All scale bars = 100um.

bioRxiv preprint doi: https://doi.org/10.64898/2026.09.14.750687; this version posted September 16, 2026. The copyright holder for this preprint (which was not certified by peer review) is the author/funder, who has granted bioRxiv a license to display the preprint in perpetuity. It is made available under a CC-BY 4.0 International license.

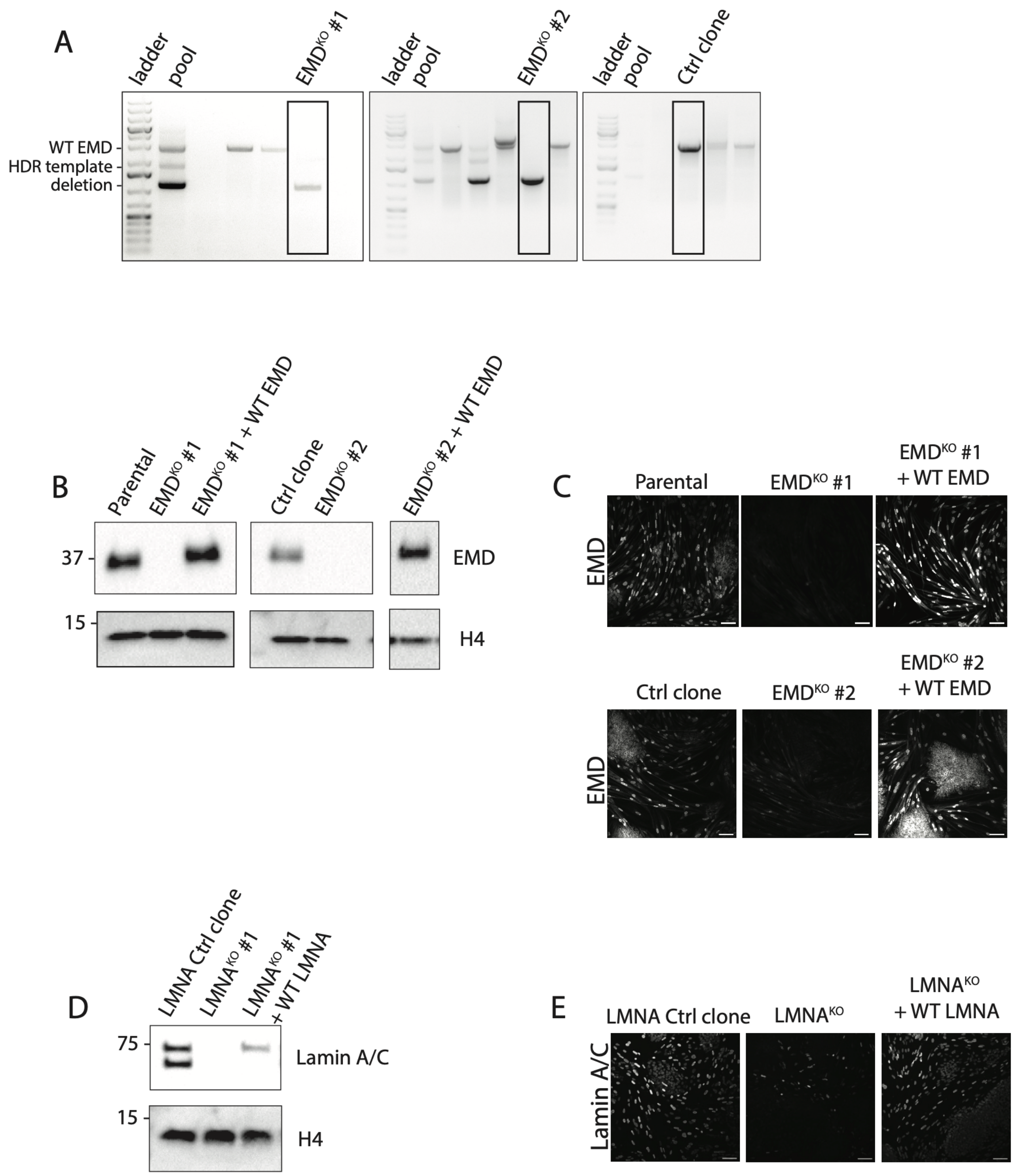

Supplementary Figure 2. Validation of *EMD* KO and *LMNA* KO hiPSCs.

A) Genotyping PCR for *EMD* KO #1, *EMD* KO #2, and CRISPR control. Upper band corresponds to wildtype emerin locus, lower band corresponds to deletion within the *EMD* locus. B) Western blot of CRISPR control clone and both *EMD* KO clones with corresponding rescue. C) Immunofluorescence staining of CRISPR control clone and both *EMD* KO clones with corresponding rescue. D) Western blot of control, *LMNA* KO, and *LMNA* KO + Lamin A rescue, blotted for Lamin A/C. E) Immunofluorescence staining of control, *LMNA* KO, and *LMNA* KO + Lamin A rescue. All scale bars = 100um.

bioRxiv preprint doi: https://doi.org/10.64898/2026.09.14.750687; this version posted September 16, 2026. The copyright holder for this preprint (which was not certified by peer review) is the author/funder, who has granted bioRxiv a license to display the preprint in perpetuity. It is made available under a CC-BY 4.0 International license.

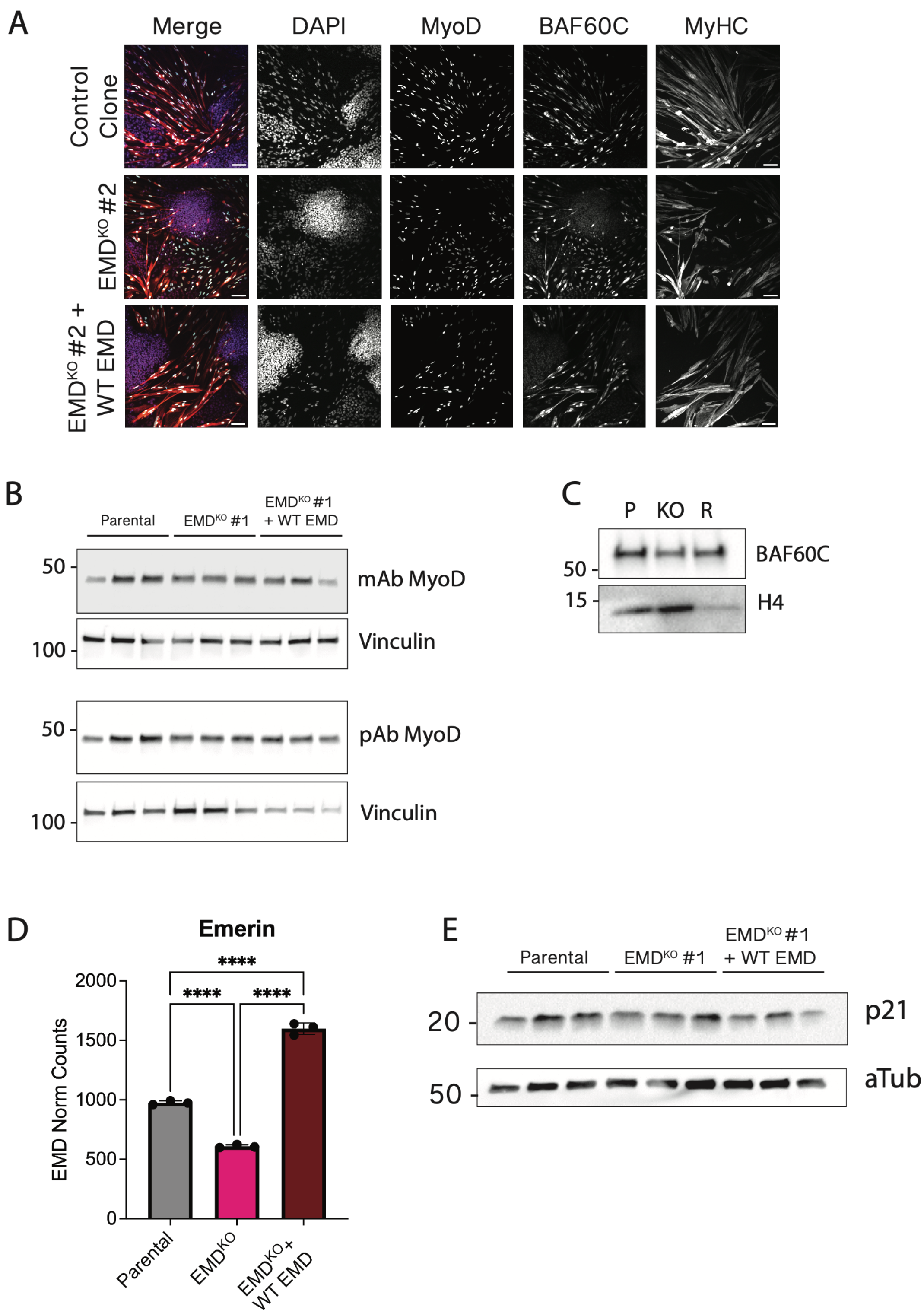

Supplementary Figure 3. Additional EMD^{KO} iSM differentiation defects and characterization of transgene, *EMD* transcript, and variable p21 levels.

(Figure caption continued on the next page.)

bioRxiv preprint doi: https://doi.org/10.64898/2026.09.14.750687; this version posted September 16, 2026. The copyright holder for this preprint (which was not certified by peer review) is the author/funder, who has granted bioRxiv a license to display the preprint in perpetuity. It is made available under a CC-BY 4.0 International license.

(Figure caption continued from the previous page.)

A) Representative images of Control, EMD KO #2, and EMD KO #2 + WT EMD Day 6 iSMs, quantified in Figure 1D. B) Three distinct biological replicates immunoblotted using mouse monoclonal (BD cat#) and rabbit polyclonal (Ptg cat#) anti-MyoD antibodies. C) Immunoblot of BAF60C levels. P = parental, KO = EMD^{KO} #1, R = EMD^{KO} #1 + WT EMD (Rescue). D) Normalized transcript counts of *EMD* in Parental, EMD^{KO} #1, and EMD^{KO} #1 Rescue. E) Three distinct biological replicates immunoblotted to demonstrate p21 variability by Western blot (compare with Figure 3E). All samples in this figure are from Day 6 iSMs. All scale bars = 100um. Proportions significantly different by one-way ANOVA: ****$p < 0.0001$.

bioRxiv preprint doi: https://doi.org/10.64898/2026.09.14.750687; this version posted September 16, 2026. The copyright holder for this preprint (which was not certified by peer review) is the author/funder, who has granted bioRxiv a license to display the preprint in perpetuity. It is made available under a CC-BY 4.0 International license.

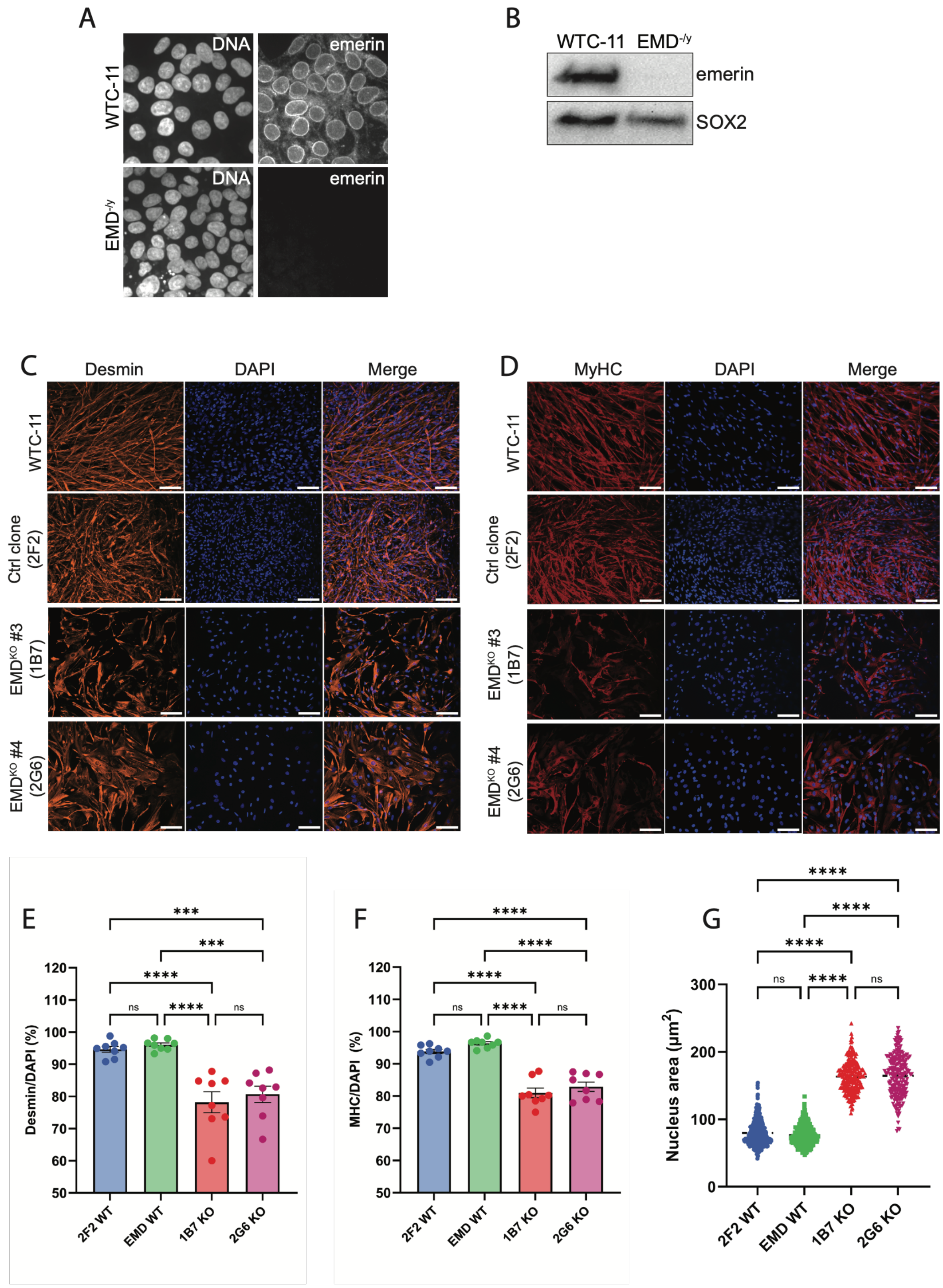

Supplementary Figure 4. Additional EMD KO clones have a skeletal muscle differentiation defect.

(Figure caption continued on the next page.)

bioRxiv preprint doi: https://doi.org/10.64898/2026.09.14.750687; this version posted September 16, 2026. The copyright holder for this preprint (which was not certified by peer review) is the author/funder, who has granted bioRxiv a license to display the preprint in perpetuity. It is made available under a CC-BY 4.0 International license.

(Figure caption continued from the previous page.)

A-B) Representative EMD-/y iPSC clone lacking detectable emerin expression by immunostaining (A) and Western blotting (B). Note preserved expression of pluripotency marker SOX2. C-D) Representative images of Desmin-positive (C) and MyHC-positive (D) skeletal muscle myotubes in WTC-11 parental hiPSCs, CRISPR control (2F2), and two *EMD* KO clones (1B7 & 2G6). E-F) Skeletal muscle differentiation efficiency scored as % of cells positive for Desmin (E) and MyHC (F) for WTC-11, CRISPR control clone (2F2), and two *EMD* KO clones (1B7 & 2G6). G) Nucleus area calculated for each nucleus within each field of view across replicates in WTC-11, CRISPR control (2F2), and two *EMD* KO clones (1B7 & 2G6). Scale bar = 100 µm. Ordinary One-way ANOVA with multiple comparisons. ***$p<0.001$. ****$p<0.0001$.

bioRxiv preprint doi: https://doi.org/10.64898/2026.09.14.750687; this version posted September 16, 2026. The copyright holder for this preprint (which was not certified by peer review) is the author/funder, who has granted bioRxiv a license to display the preprint in perpetuity. It is made available under a CC-BY 4.0 International license.

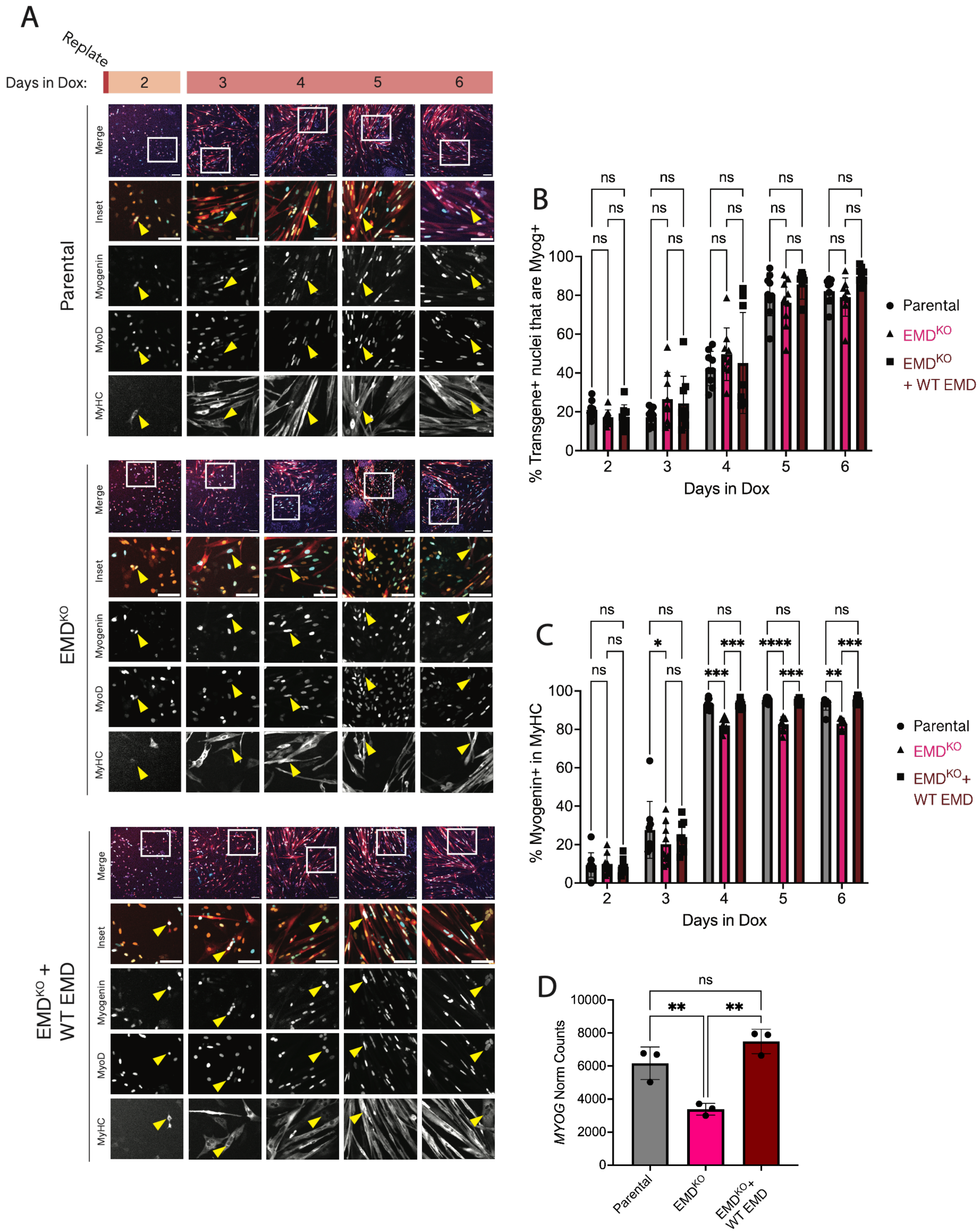

Supplementary Figure 5. Myogenin expression is decreased in EMD KO iSMs.

(Figure caption continued on the next page.)

bioRxiv preprint doi: https://doi.org/10.64898/2026.09.14.750687; this version posted September 16, 2026. The copyright holder for this preprint (which was not certified by peer review) is the author/funder, who has granted bioRxiv a license to display the preprint in perpetuity. It is made available under a CC-BY 4.0 International license.

(Figure caption continued from the previous page.)

A) Time course of iSM differentiation, co-stained for myogenin, MyoD, and MyHC. Yellow arrowheads indicate transgene+/Myog+ population, quantified in (B). Quantification of transgene-positive nuclei that are also myogenin-positive (n = 3 biological replicates per genotype). C) Quantification of myogenin-positive nuclei within MyHC-positive myotubes in (A) (n = 3 biological replicates per genotype). D) Normalized transcript counts of *MYOG*. All scale bars = 100um. Proportions significantly different by two-way ANOVA in (B) and (C), one-way ANOVA in (D): $^{**}p < 0.01$, $^{***}p < 0.001$, $^{****}p < 0.0001$.

bioRxiv preprint doi: https://doi.org/10.64898/2026.09.14.750687; this version posted September 16, 2026. The copyright holder for this preprint (which was not certified by peer review) is the author/funder, who has granted bioRxiv a license to display the preprint in perpetuity. It is made available under a CC-BY 4.0 International license.

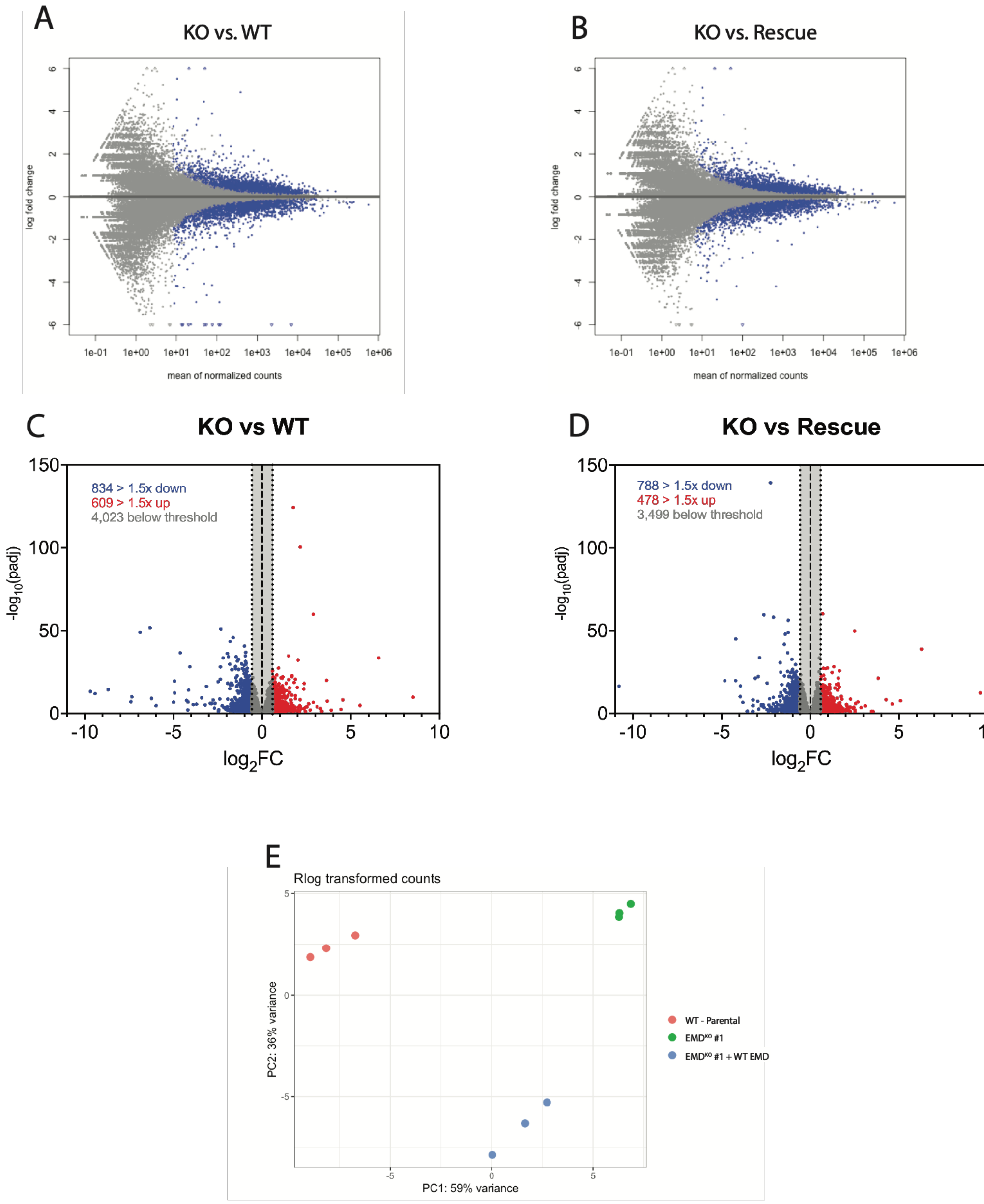

Supplementary Figure 6. Analysis of transcriptional changes after EMD KO in Day 6 iSMs.

A-B) MA plots comparing gene expression in KO vs. WT (A) and KO vs. Rescue (B). 5,466 differentially expressed genes in (A) and 4,765 in (B) with p-adj < 0.05 shown in blue. C-D) Log_2FC of differentially expressed genes shown in (A) and (B), plotted against -log(p-adj). 609 genes are upregulated > 1.5-fold, 834 downregulated > 1.5-fold in (C). 478 genes are upregulated > 1.5-fold, 788 downregulated > 1.5-fold in (D). E) Principal component analysis (PCA) plot for three biological replicates across the three genotypes analyzed by RNA-seq.

bioRxiv preprint doi: https://doi.org/10.64898/2026.09.14.750687; this version posted September 16, 2026. The copyright holder for this preprint (which was not certified by peer review) is the author/funder, who has granted bioRxiv a license to display the preprint in perpetuity. It is made available under a CC-BY 4.0 International license.

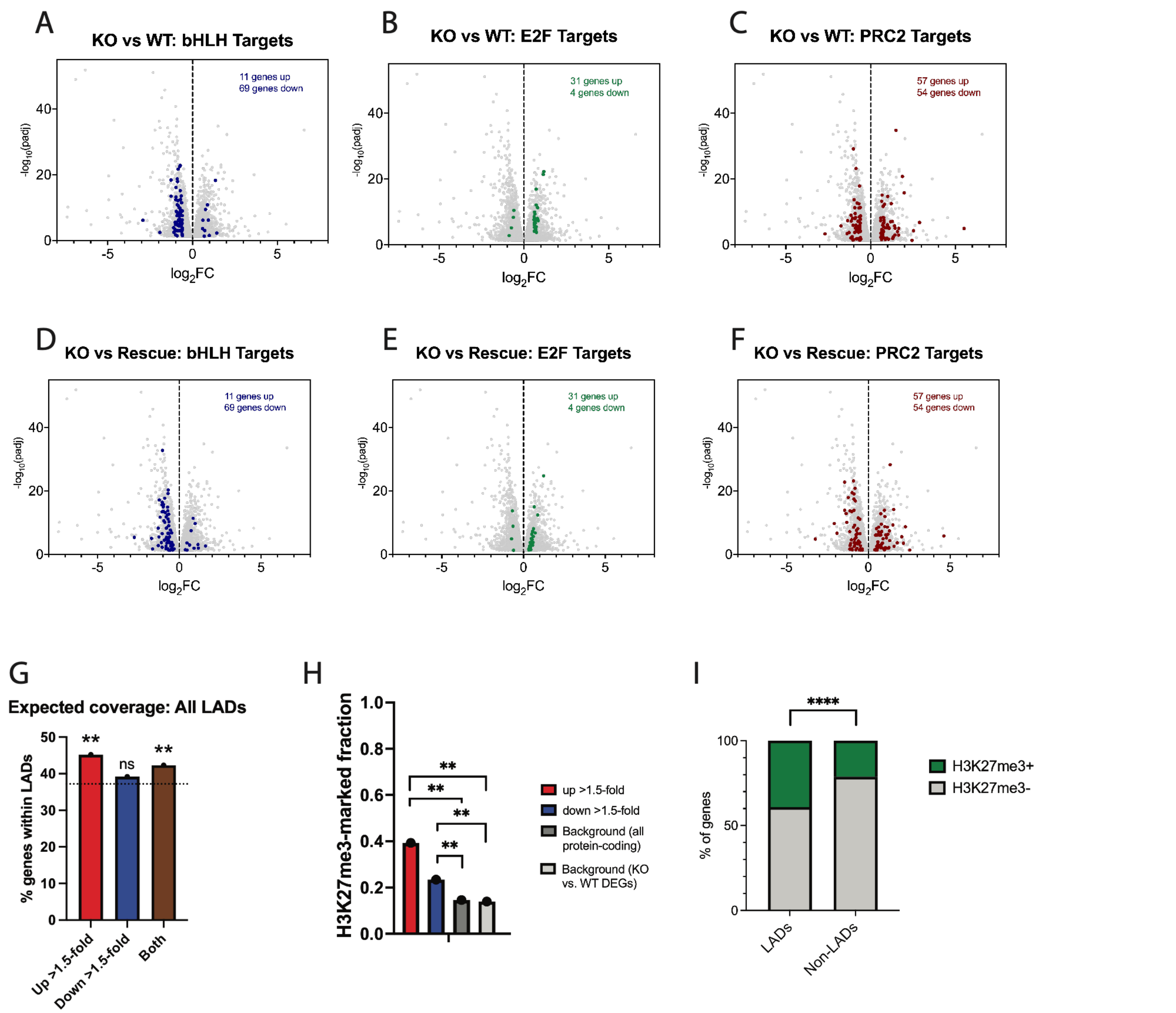

Supplementary Figure 7. Analysis of rescue-sensitive gene distribution among transcription factor targets, LADs, and H3K27me3-positive regions.

A-F) Log2FC of differentially expressed genes between KO vs. WT (A-C) and KO vs. Rescue (D-F), plotted against -log(p-adj). Gene targets of bHLH TFs (A & D), E2F TFs (B & E), and the PRC2 complex (C-F) are highlighted. G) LAD enrichment among up and downregulated rescue-sensitive genes, or the union of both. Dotted line represents background (average LAD coverage across all human protein-coding genes). H) Fraction of genes per set predicted to be H3K27me3-

(Figure caption continued on the next page.)

bioRxiv preprint doi: https://doi.org/10.64898/2026.09.14.750687; this version posted September 16, 2026. The copyright holder for this preprint (which was not certified by peer review) is the author/funder, who has granted bioRxiv a license to display the preprint in perpetuity. It is made available under a CC-BY 4.0 International license.

(Figure caption continued from the previous page.)

marked in human myotubes. Up and downregulated rescue-sensitive genes were compared to both an all protein-coding gene background and the full DEG list of KO vs. WT background. I) Percentage of all genes within LADs and non-LADs that are predicted to be H3K27me3-marked in human myotubes. All percentages significantly different when compared to background(s) by two-sided Fisher's exact test: *$p < 0.05$, **$p < 0.01$, ***$p < 0.001$, ****$p < 0.0001$.

bioRxiv preprint doi: https://doi.org/10.64898/2026.09.14.750687; this version posted September 16, 2026. The copyright holder for this preprint (which was not certified by peer review) is the author/funder, who has granted bioRxiv a license to display the preprint in perpetuity. It is made available under a CC-BY 4.0 International license.

Supplementary Table 1. List of RT-qPCR and genotyping primers.

**Target**	**Forward sequence**	**Reverse sequence**
mMyoD (Caputo et al. 2020)	AATGGCTACGACACCGCCTACTA	AGATGCGCTCCACTATGCTGGACA
hBAF60C (Caputo et al. 2020)	GCGCGCAAAGCCACGAAA	ATCCGGGCTCCAGACGGCATC
hEMD	CTCGGCCGGTTTTGGTAG	GTCAGCTCGGTATCCGAAAG
hB3GAT1	GCAGCCTCCTTCGAGAACTT	AGCCCTTCTTGCCCTCATTC
hSox2	GGGAAATGGGAGGGGTGCAAAAGAGG	TTGCGTGAGTGTGGATGGGATTGGTG
hMyogenin (Caputo et al. 2020)	AATGCAGCTCTCACAGCGCCTC	TCAGCCGTGAGCAGATGATCC
hGAPDH	CGCTCTCTGCTCCTCCTGTT	CCATGGTGTCTGAGCGATGT
hEMD (genotyping)	GGGGAACCCTAGGCTCTG	GGGAGCAAGCACTACTCTGG

bioRxiv preprint doi: https://doi.org/10.64898/2026.09.14.750687; this version posted September 16, 2026. The copyright holder for this preprint (which was not certified by peer review) is the author/funder, who has granted bioRxiv a license to display the preprint in perpetuity. It is made available under a CC-BY 4.0 International license.

Supplementary Table 2. List of antibodies.

Target	Isotype	Application	Dilution	Catalog No.
MyoD	Mouse IgG1	IF	1:150	BD 554130
		IP	1:120	
		WB	1:1000	
MyoD	Rabbit	IF	1:400	Proteintech 18943-1-AP
		WB	1:2000	
BAF60C	Rabbit	IF	1:100	Proteintech12838-1-AP
		WB	1:1000	
MyHC	Mouse IgG2b	IF	1:50	DSHB MF 20
		WB	1:100	
EMD	Rabbit	IF	1:300	Proteintech 10351-1-AP
		WB	1:2000	
p21	Rabbit	IF	1:400	Cell Signaling Technology 2947T
		WB	1:1000	
Myogenin	Mouse IgG1	IF	1:50	DSHB F5D
Vinculin	Mouse IgG1	WB	1:4000	Sigma-Aldrich V9264-100UL
LMNA	Mouse IgG2a	IF	1:500	Active Motif 39288
		WB	1:1000	
Histone H4	Rabbit	WB	1:1000	Active Motif 39070
Pan-acetyl-K	Rabbit	WB	1:1000	Cell Signaling Technology 9814S
E2A	Rabbit	WB	1:1000	Cell Signaling Technology 4865S
Desmin	Mouse IgG1	IF	1:100	Dako/Agilent M0760
